# Radiodontia—Its Uses and Misuses

**Published:** 1922-06

**Authors:** Herbert G. Frankel

**Affiliations:** U. S. A. Dental Surgeon, Camp Taylor, 1917-1919, Member Staff Jewish Hospital, 4 W. 7th Street, Cincinnati, O.


### RADIODONTIA—ITS USES AND MISUSES

BY HERBERT G. FRANKEL, D.D.S.

U. S. A. Dental Surgeon, Camp Taylor, 1917-1919, Member Staff Jewish Hospital.

When Roentgen discovered the X-ray little did he dream that it would have the great value in preventive medicine and dentistry that it has today, or that it would almost revolutionize the two great sciences.

Just let us look at its varied uses. It has great diagnostic value, and practically all the diseases of tissue or bone are diagnosed by the X-ray. Then it is used in the treatment of a great many skin and organic diseases, etc.

In dentistry its uses are for diagnosis of focal infection, peridontal disease and other conditions, such as impacted teeth, fractured jaws, etc. It has become indispensable to the modern practitioner and every up-to-date office contains this valuable equipment. Those who do not have this equipment, and need X-rays in their work, send it out to some dental or medical X-ray laboratory and depend on the diagnosis of this laboratory to a great extent.

Now, lets get down to the real issue, and talk of the misuses of the X-ray. First of all I want to say that the greatest misuse of the X-ray is caused by faulty technic. I, myself, have been doing X-ray work for about five years, and I still believe that I can improve a great deal in my technic. Angles are most important, but the most important thing is to get more than just one view of a single tooth. The difference in the various pictures will show you how important this is. Secondly, bone penetration in different individuals is different, and calls for longer or shorter exposures, as the case may be. Where you have heavy, dense-boned individuals, you have to give longer exposures, but sometimes the bone is very dense in individuals who you do not suspect. I believe that it is well to vary exposures, and take two or more pictures at different angles and vary the time, from 2 to 4 seconds with Xtra Fast Films. In this manner you will be able to avoid blotting out the detail, which is the most important thing.

Thirdly, the developing and fixing of pictures is an art that comes only with the finest of technic. I have seen wonderful films actually spoiled, by poor developing and fixing. The first thing necessary is a real dark room, not some little cubby hole, where you have to come up for air every two minutes, but a place large enough to work in. Developer must be fresh and not a month old. Summer is the worst time of the year for results in radiography and well do we know what it means when we do not keep our developing agents down to 65 degrees C. in warm weather. Ice is our only salvation and a good electric fan to dry our films so that they do not run and spoil our work. In developing do not get the film to dark or too light, but there is a happy medium, and this can be found by constant practice. Eastman says develop 5 minutes at 65 degrees C., and this usually brings results, but sometimes it requires a little more time than that.

Now comes diagnosis, and this is the most important of all, and how often is the X-ray misused here. Every shadow a focal infection, and every spot a pathological condition. I know what you are going to say, "That is a lie," but my dear colleagues, back just a few years, when the great cry was, "Take them all out," we might have overlooked some and saved them, but we did not. I believe the dental practitioner makes less mistakes today in diagnosing radiographs than any other profession, but I still think that we can improve. By taking different views of a case, we can get a better prospective. I believe that every dentist studying radiographs should have a radiographic picture of healthy bone and one of diseased bone before him, so that he can differentiate between them in making his decision, as his decision is like that of a judge, who must decide whether the prisoner is guilty or not.

PART TWO

It gives me great pleasure now to take up what I consider the greatest misuses of the X-ray and the causes.

First of all, I am going to take a rap at our own profession, and especially at those men who are trying to do every kind of X-ray work, including treatment. I feel that we as dentists have enough to do in our own pastures, without tramping over the fertile soil of medicine, and unless a man is qualified with a good foundation in the pathology of the entire body, and can recognize these conditions, I believe that he will be better off if he makes himself proficient in his own line and does not try to take in too much territory. And for him to treat the various skin and organic diseases, well, it is all right if he can get away with it, but it would be better if he would devote his time to his dental work, as he would get more out of it in the end.

Now, as to physicians doing dental work and making dental diagnosis, is just as bad and even worse than the dentists doing medical work, because in the first place the average physician knows little or nothing about dental pathology and most of them care less, but every day in the week you will see some physician who will send a patient to another physician who is a radiographer and have him take a full mouth X-ray, and when the report comes back it is usually full of rarefactions and bone degenerations and the patient is then told that to save her life she must have her teeth out. Now, don't think I am radical, but suppose that the same patient should go to the dentist for a toothache and the dentist sends her to one of his colleagues to have her appendix rayed and without even consulting the surgeon, should say that you must have your appendix out. See how foolish that is, but that is what is happening every day among our M.D.'s.

Physicians will do more good if they will send their patients to a dentist, and if the dentist deems it necessary to have radiographs, and then to extract the teeth, he should be the one to decide.

Lastly, the X-ray laboratory is run like a dental laboratory, a pathological laboratory or any other laboratory. Sometimes a physician or dentist is at the head, and again, someone who has done a great deal of that kind of work, and who can get the work out in fairly good shape. Now, I am not knocking every laboratory, nor do I wish to infer that the work that some of them turn out is not the best, because I have seen some fine work in these laboratories, but to get back to the sledge, I have also seen some very punk work, too. Now, I am not in favor of allowing some assistant to grind out pictures, and especially one who has never studied any pathology. Moreover, I would not want such a person to treat me with an X-ray any more than I would want anyone but the best surgeon to operate on me. Then I would hate to have to accept as final the judgment as to diagnosis, made by this same individual.

Most of the profession accept the report of these laboratories, without question. Also the individual attention that you could give that patient, at your own office, would be very costly to the patient at an X-ray laboratory. Now do not think that I am trying to say that the dental X-ray laboratories are very much more expensive than the individual offices, as they are not, but in your own office you would probably take two or more pictures if you wanted to make a diagnosis, but if you sent it out you would probably only ask for one. The dental X-ray laboratory is a necessity for the man who can not afford a dental machine of his own, but the man who uses this method should also study diagnosis to a great degree and not depend on the other individual. As long as I have been doing this work

I have never voluntarily given a dentist a report, and only when he asked my opinion, would I state my views. Some men are wonderful diagnosticians and they can perceive things that would puzzle you greatly.

In conclusion, I would state that the X-ray is a wonderful discovery for mankind and the only danger that confronts us is the fact that it may become a fad and carried too far.

My advice is to use it cautiously, but use to the best advantage in medicine and dentistry. Let each profession keep to their own field and learn its value there.

4 W. 7th Street, Cincinnati, O.

